# A cloud-based training module for efficient *de novo* transcriptome assembly using Nextflow and Google cloud

**DOI:** 10.1093/bib/bbae313

**Published:** 2024-06-28

**Authors:** Ryan P Seaman, Ross Campbell, Valena Doe, Zelaikha Yosufzai, Joel H Graber

**Affiliations:** MDI Biological Laboratory, 159 Old Bar Harbor Road, Bar Harbor, ME 04609, USA; Health Data and AI, 4301 Fairfax Dr, Unit 210, Deloitte Consulting LLP, Arlington, VA 22203, USA; Google Cloud, Google, 1900 Reston Metro Plaza, Reston, VA 20190, USA; Health Data and AI, 4301 Fairfax Dr, Unit 210, Deloitte Consulting LLP, Arlington, VA 22203, USA; MDI Biological Laboratory, 159 Old Bar Harbor Road, Bar Harbor, ME 04609, USA

**Keywords:** cloud computing, nextflow, training module, transcriptome, assembly, annotation

## Abstract

This study describes the development of a resource module that is part of a learning platform named “NIGMS Sandbox for Cloud-based Learning” (https://github.com/NIGMS/NIGMS-Sandbox). The overall genesis of the Sandbox is described in the editorial NIGMS Sandbox at the beginning of this Supplement. This module delivers learning materials on *de novo* transcriptome assembly using Nextflow in an interactive format that uses appropriate cloud resources for data access and analysis. Cloud computing is a powerful new means by which biomedical researchers can access resources and capacity that were previously either unattainable or prohibitively expensive. To take advantage of these resources, however, the biomedical research community needs new skills and knowledge. We present here a cloud-based training module, developed in conjunction with Google Cloud, Deloitte Consulting, and the NIH STRIDES Program, that uses the biological problem of *de novo* transcriptome assembly to demonstrate and teach the concepts of computational workflows (using Nextflow) and cost- and resource-efficient use of Cloud services (using Google Cloud Platform). Our work highlights the reduced necessity of on-site computing resources and the accessibility of cloud-based infrastructure for bioinformatics applications.

## Introduction

The advent of cloud computing has made possible broad new avenues of computational analysis at cost-effective prices. Effective use of these resources necessitates new knowledge and skills, and accordingly user-friendly training materials. We present a cloud-based training module that has three primary learning goals: (i) from a *biology perspective*, demonstration of the process of transcriptome assembly from raw RNA-seq data, (ii) from a *computational perspective*, demonstration of carrying out complex, multi-step computations using workflow management and container systems, and (iii) from an *infrastructure perspective*, demonstration of carrying out these analyses efficiently in a Cloud environment through use of an API that automatically provisions, uses, and then releases needed computational resources on demand.

### The biological challenge: assembling a transcriptome from short read sequencing data

The advent of next-generation sequencing (NGS) technologies introduced novel ways to study molecular biology [1]. The accessibility and affordability of these technologies have made gene expression studies through transcriptome profiling analysis, primarily in the form of short-read RNA sequencing (RNA-seq), a standard tool for the molecular characterization of biological systems [2]. A ‘transcriptome profile’ is a measurement of which gene’s transcripts are present and in what abundance in each sample. Comparison of transcriptome profiles between samples (called differential expression analysis) allows identification of the molecular differences between them, revealing systematic effects of perturbation, e.g. mutation versus wildtype or treated versus control.

Well-studied model organisms like mouse, human, and zebrafish have the essential resources available for the analysis and interpretation of the collected data (i.e., well-assembled genomes and large collections of annotated transcripts). In contrast, less well-studied non-model organisms present a challenge in that these critical resources often do not exist or are in elementary stages of development. Despite their lack of resources, these less-studied organisms can provide unique experimental systems with potential for translation to human health and welfare such as better understanding the mechanisms of aging or the effects of early life stress. To work with expression data from these organisms, these critical resources must often be created before new data can be analyzed.

RNA-seq data analysis commonly involves alignment to a genome followed by assignment to transcripts and genes. For organisms with well-annotated transcriptomes, computational resources can be saved by aligning the data directly to the transcriptome. In the absence of a genome or well annotated transcriptome, a *de novo* assembled transcriptome (generated from the same RNA-seq reads that will subsequently be used for expression profiling) can be used as an alignment target for mapping RNA-seq reads to estimate gene and transcript abundance [3].

**Figure 1:**
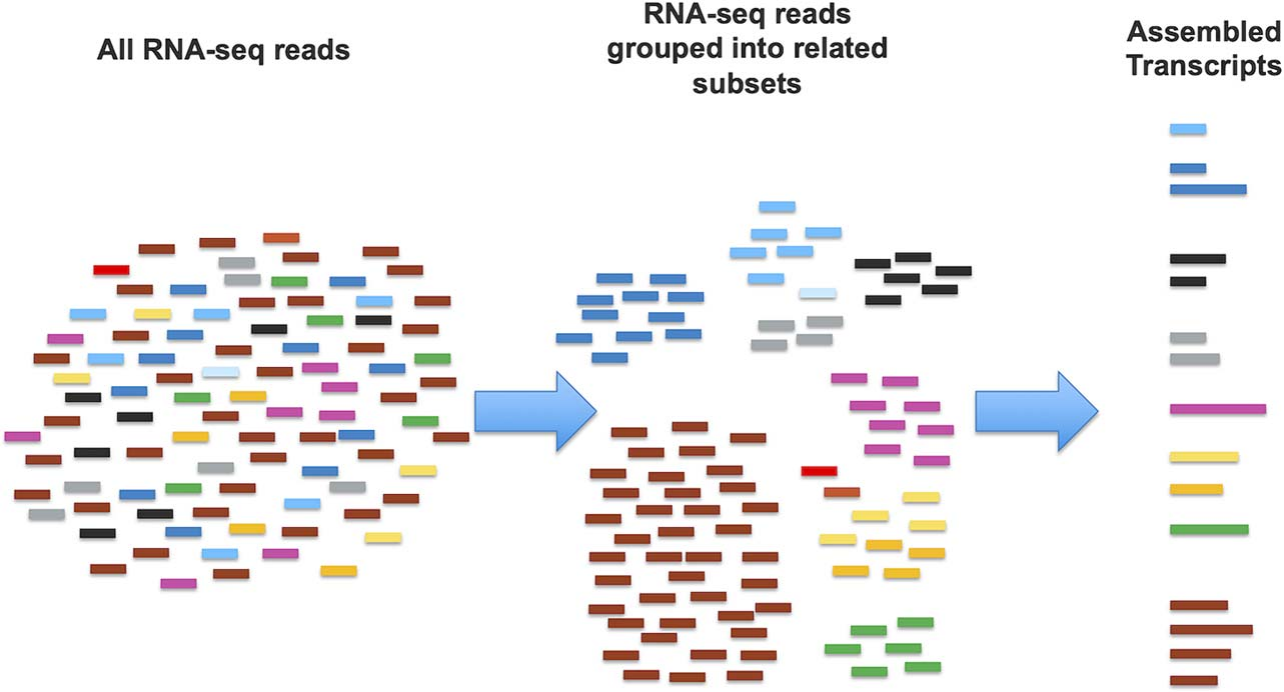
Conceptual steps of *de novo* transcriptome assembly. Short RNA-seq reads (at left, with colors representing the unknown gene/transcript of origin) are first segregated into groups of reads that are judged likely to have derived from a single gene (center). Each cluster is subsequently analyzed to determine the most likely underlying transcripts (right).

*De novo* transcriptome assembly is the process of constructing a working version of an organism’s transcriptome based on the large-scale but fragmentary data provided by RNA-seq data. In brief, the process of assembling a transcriptome from raw reads (Fig. 1) is to first make a ‘best guess’ segregation of the reads into subsets that are most likely derived from one (or a small set of related/similar genes), and then for each subset, use the grouped reads to construct a most-likely set of transcripts and genes [3].

Several popular computational tools have been developed for assembling transcriptome data [4–8], each with known strengths and weaknesses [3, 9]. Meta-analysis suggests that combination of predictions from multiple programs (and possibly ranges of input arguments) will create a more comprehensive and accurate transcriptome [10, 11]. TransPi [10] the workflow used in this module (described below), explicitly takes this approach, creating multiple assemblies, integrating, and subsequently refining these into a unified final transcriptome.

**Figure 2:**
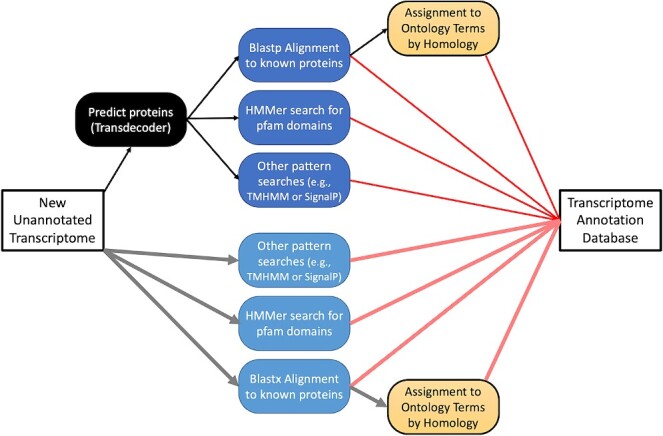
A conceptual workflow for annotation on a new, unannotated transcriptome.

Once a new transcriptome is generated, assessed, and refined, it must be annotated with putative functional assignments to be of use in subsequent functional studies. Functional annotation is accomplished through a combination of assignment of homology-based and *ab initio* methods. The most well-established homology-based processes combine protein-coding sequence prediction with subsequent protein sequence alignment to databases of known proteins, especially those from human or common model organisms (Fig. 2). In contrast, *ab initio* methods use computational models of various features (e.g., known protein domains, signal peptides, or peptide modification sites) to characterize either the transcript or its predicted protein product.

### Computational efficiency: managing complex analysis with workflow systems

While *de novo* transcriptome assembly and annotation is more feasible and less resource intensive than genome assembly, it is still a complex task with numerous computationally demanding steps. It is standard practice in computational biology and other fields that such complex analyses are carried out not by a single comprehensive program, but instead by a defined sequence of multiple programs, in which outputs of earlier steps become inputs to later steps. The process of systematically executing these steps in the proper order with proper passing of data between steps is collectively called a *workflow* or *pipeline*.

**Figure 3:**
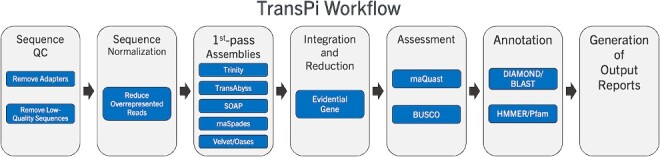
TransPi [10] workflow for a basic transcriptome assembly run.

As noted, this learning module uses the TransPi workflow [10], which implements multiple computational tools in a systematic manner. TransPi starts with one or more RNA-seq data files as the primary input and runs through the following stages: (i) Preprocessing including quality control, (ii) Assembly, using multiple computational approaches, (iii) Integration and reduction of preliminary assemblies, (iv) Assessment and Refinement of the integrated assembly, and (v) annotation of the final assembly. The components of this workflow are shown graphically in Fig. 3.

Workflow management systems, e.g. Nextflow (https://www.nextflow.io), provide a syntax for defining the order of steps and the associated flow of information between steps, while also providing platform-specific control software that can read and execute these workflows in an efficient and cost-effective manner [12]. The workflow control systems are responsible for allocating resources, activating and executing analysis steps, and ensuring that all steps occur in the proper order (e.g. only activating a sequence alignment program after the sequence quality control has been performed).

TransPi [10] was implemented in the Nextflow language, which controls the information flow, orchestrates the order in which specific processes are executed, and finally organizes the output. Nextflow also uses containers (Docker, Singularity) or conda environments to manage the dependencies for the specific software used for individual processes.

### Using cloud resources efficiently with google batch

Transcriptome assembly is well tuned for the use of Cloud resources, due to the frequently large computational requirements. While this module is designed to use a small test dataset that can be run with modest resources, analysis of a full data set can require large scale computing resources including tens to hundreds of processors and hundreds of gigabytes of memory.

While the training data set provided with this module is small enough to run in a single computing instance, specifically a Vertex AI Jupyter notebook, any real-world full-sized data set would require significantly more computational resources. As such, we have included instructions in this module for interfacing with and utilizing Google Batch, a service provided by Google that both allows for batch job scheduling and managed compute infrastructure.

**Figure 4:**
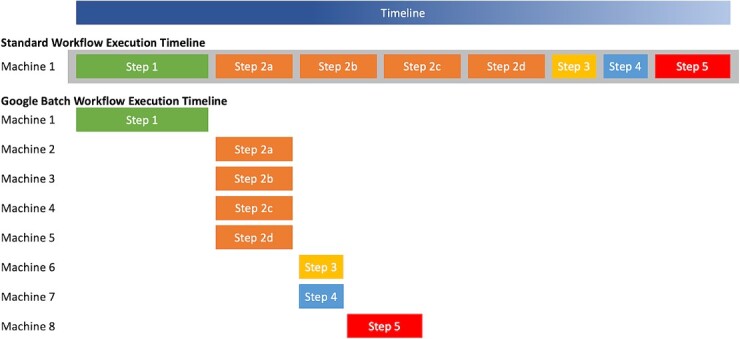
A conceptual illustration comparing execution of a Nextflow pipeline in the standard method (on a single machine) and on Google batch (on distributed machines).

The key to cost-efficient cloud computing is to only use the resources you need for as long as you need them. Google Batch allows us to control our process from a modest, inexpensive machine that can interface with GCP to provision and use the more expensive machines needed for computing, minimizing costs by terminating and releasing resources as soon as the computations for a specific step are finished and the results are safely copied to persistent storage. Individual steps have a predefined compute requirement, reducing wasted cost on underutilized compute. Processing steps that are independent of each other can be run in parallel on separate (typically virtual) machines (Fig. 4). Finally, Nextflow explicitly supports Google Batch as a compute environment, mapping computational tasks onto GCP computing resources and running them in an efficient manner.

### Overview of this training module

We developed a cloud-based learning module to leverage GCP to educate researchers about *de novo* transcriptome assembly and the benefits of utilizing Cloud computing platforms for their own research. The objective of this learning module is to teach the basics behind the biology, the computational processes, and the implementation of the computational tools required to build a transcriptome. This module includes two introductory submodules (Submodule_00_background.ipynb and Submodule_00_Glossary.md) to bring the users up to speed, giving an overview, background, and a summary of the materials to be covered in the remaining modules. The next submodule (Submodule_01_prog_setup.ipynb) guides users through the setup steps needed for carrying out the subsequent analysis in a VertexAI Jupyter notebook. The next module (Submodule_02_basic_assembly.ipynb) carries out the steps of a basic assembly using the small test data set. The penultimate submodule (Submodule_03_annotation_only.ipynb) carries out a larger analysis focused only on the downstream steps of annotating and understanding the transcriptome. The final submodule (Submodule_04_google_batch_assembly.ipynb) introduces the use of Google Batch, carrying out the same analysis as in Submodule 2, but in a distributed manner, giving users the skills to apply to other problems.

We recommend that users follow the submodules in order as they build on each other. Note that this module was built specifically assuming GCP as the provider of cloud resources. All setup from the cloned GitHub repository is specifically designed for execution within Jupyter notebooks provisioned by a GCP Vertex AI instance. With various adjustments to setup and commands within the submodules, the module could be adapted to run on a different platform such as Amazon Web Services (AWS) or Microsoft Azure. The primary difference would be in adapting the Nextflow config file to point to a preconfigured AWS Batch or Azure Batch environment.

## Method and implementation

### Technical requirements

**Figure 5:**
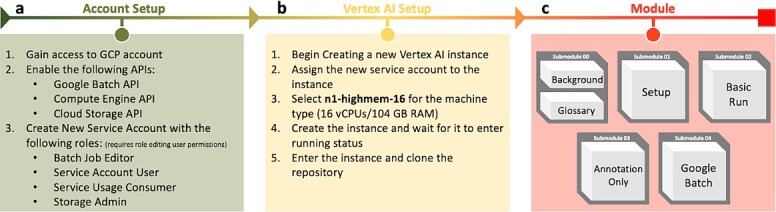
A diagram showing the aspects of the module, including account setup, vertex AI setup, and finally module execution.

Google Cloud Platform (GCP) is a powerful service providing more than 100 distinct services to their users, however specific services need to be enabled for use them within their account. This module requires the Google Batch API, Compute Engine API, and Cloud Storage API. Additionally, for security purposes, GCP restricts access to users and services within your account preventing access unless explicitly given permission. For successful execution of submodule 04, a service account needs to be created for Nextflow that has the following roles: Batch Job Editor, Service Account User, Service Usage Consumer, and Storage Admin. The user must either have the proper permissions to request or grant these roles to a service account (Fig. 5a).

When setting up the Vertex AI instance for this module, the Nextflow service account must be assigned to that instance. Due to the high computational requirements of transcriptome assembly and the associated processes within the TransPi workflow, the Vertex AI instance within GCP needs to be provisioned accordingly. During instance setup, the Machine Type should be set at n1-highmem-16 which provides 16 vCPUs and 104 GB of RAM. Note: this is a relatively expensive class of machine to run, so it should be paused or deleted when not in use. For the last submodule, we provide instructions to substantially reduce the requested resources, since workflow processes are submitted and executed on individual instances provisioned by Google Batch. The module will need to be downloaded into the Vertex AI Jupyter lab environment using a git command (Fig. 5b). Instructions and guidance for this process are included in the README file associated with our GitHub repository.

## Module details

**Figure 6:**
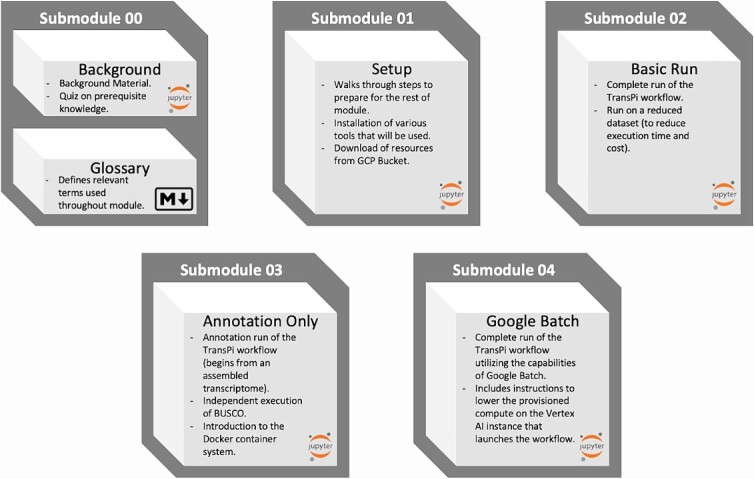
A visual breakdown of the submodules.

All of the submodules (shown with visual summaries in Fig. 6) include various progress quizzes that check the user’s comprehension of the material along with periodic videos to further explain the biology and processes that are being carried out.

### Submodule 00 background and glossary

The background submodule (Submodule_00_Backrgound.ipynb) is a Jupyter notebook that covers background material relevant to this module. It also includes a brief quiz at the beginning to check baseline knowledge regarding ideas of DNA, RNA, transcription, and gene regulation. The glossary submodule (Submodule_00_Glossary.md) is a markdown file that defines relevant terms used throughout the module.

### Submodule 01 setup

The setup submodule (Submodule_01_prog_setup.ipynb) is a Jupyter notebook that must be run to establish the environment required for the rest of the module. Steps in this submodule include installation of several software packages (e.g., Java, Nextflow, Mamba, and the TransPi workflow) and subsequent download and organization of required resources (databases, programs, and configuration files) from a dedicated GCP bucket.

### Submodule 02 basic run

The basic run (Submodule_02_basic_assembly.ipynb) is a Jupyter notebook that carries out a complete run of the Nextflow TransPi assembly workflow. It uses a modest sequence set, producing a small assembled transcriptome. Given that this is a small dataset, the transcriptome assemblies and integration will run in a reasonably short time, however several of the downstream analyses are designed for complete (or nearly so) transcriptomes, and as such, their output will not be informative, providing the motivation for Submodule 03.

### Submodule 03 annotation only run

To better understand the annotation steps, we have taken advantage of TransPi’s capacity to run in ‘annotation only’ mode, which takes an already assembled transcriptome and carries out the homology and protein domain searches, providing an example output that users can inspect with more realistic results than from the small test data set from Submodule 01.

In addition to the annotation only run, we have also provided instructions for an independent execution of the BUSCO (Benchmarking Universal Single-Copy Orthologs) analysis [13, 14], since this step is not included in the ‘Annotation Only’ option of TransPi. BUSCO analysis [15] is a standard approach for assessing the completeness and accuracy of a genome or transcriptome assembly. In short, BUSCO analysis assesses the presence and multiplicity of genes that have been identified to be nearly universally present as single copy across defined phylogenetic ranges.

Independent execution of BUSCO also allows us to give a basic introduction and rudimentary skills for working with compute container systems. Specifically, execution of the BUSCO analysis is facilitated through a publicly accessible Docker container. Users are guided through the process of acquisition and execution of the BUSCO container, providing the basic skills and understanding that can be subsequently applied in other computing contexts.

### Submodule 04 google batch run

**Figure 7:**
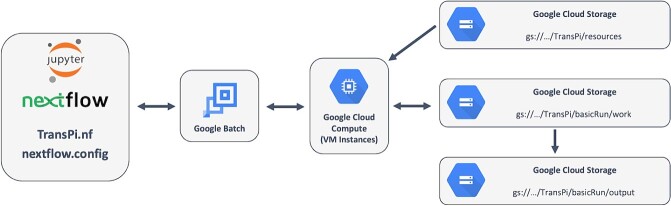
Diagram illustrating the interactions between the components used for the Google batch run.

The Google Batch run (Submodule_04_google_batch_assembly.ipynb) is a Jupyter notebook that runs through the same analysis from Submodule 02, but leveraging the computational capabilities of Google Batch (Fig. 7 shows a diagram of necessary communications for carrying out this process.). The beginning of the submodule includes instructions for downsizing the Vertex AI instances because the computation is no longer being done on the instance itself.

## Results

### Datasets

The test dataset used in submodules 02 and 04 are a downsampled version of a *Danio rerio* RNA-seq dataset (SRA database Bioproject: PRJNA318296). This data was originally generated by Hartig *et al*. [16]. Downsampling was implemented for this module to streamline the performance and accelerate the progression through the module as these are long, memory-intensive processes. While these datasets facilitate a modest execution time for assembly, their assembled transcriptomes are too small to give meaningful results in the annotation and evaluation steps.

For submodule 03, three already-assembled transcriptomes are originally obtained through the NCBI Transcriptome Shotgun Assembly archive, each with specific characteristics to highlight and each large enough to give meaningful results in these analyses.

- *Microcaecilia dermatophaga*: Originally generated by Torres-Sánchez *et al.* [17] (Bioproject: PRJNA387587).
- *Oncorhynchus mykiss*: Originally generated by Wang J et al., 2016 [18], Al-Tobasei *et al*. [19], and Salem *et al.* [20] (Bioproject: PRJNA389609).
- *Pseudacris regilla*: Originally generated by Laura Robertson, USGS (Bioproject: PRJNA163143).

The listed datasets are copied down into the Vertex AI Jupyter lab environment from a curated GCP bucket specifically for this module.

### Benchmarking info

While not included as part of the module, testing was conducted on a larger dataset to give a more realistic time and cost breakdown. Three complete zebrafish samples from the same experiment used in submodules 02 and 04 were run through the TransPi pipeline, using an n1-highmem-32 type instance, which has 32 vCPUs and 208 GB of RAM. This analysis took a total of 33 hours and 41 minutes and cost $44 for the compute ($1.3011/hour).

### Challenges and pitfalls

This module was designed to be easy to follow with minimal places for things to go wrong. The following are suggestions and recommendations if difficulties are encountered:

- All code cells within each submodule must be executed in order and must also be run in the proper directory. Directories are set at the beginning of each submodule, however, users might inadvertently change these locations.
- If the quizzes embedded within the submodules are unable to load, the user should ensure that the *pip install…* commands were run at the beginning of Submodule 00.
- Enabling the proper APIs is important for this module to work properly. To ensure there are enabled, the user can go to the API library page within the GCP console and search for the required APIs. Selection of the specific API will open a page that indicates if the API is enabled.
- Service accounts are a key feature of GCP that help protect accounts from undesirable and/or unintended actions. The ability to grant roles to service accounts is restricted to those that have been permitted by the owner of the GCP account. If the user cannot add roles to the service account, the owner/administrator should be consulted for access to these permissions.
- It is essential that the Service Account is created, given the proper roles, and added to the Vertex AI instance during setup before the instance is fully created. If this is not done, the submodules will not work properly, especially Submodule 04. Errors regarding the inability to create a new Google Bucket and new launch VM instances (within the Google Batch run) are indicative of insufficient Service Account roles.

### Goal of the training module

Our primary focus in this module was to use the biological problem of *de novo* transcriptome assembly and analysis as a means of introducing and giving basic experience with workflows, specifically Nextflow, and with Cloud computing through an API, specifically Google Batch. The power of using a predefined workflow is that for a modest user effort, a large set of related and well-organized output is generated.

Execution of a Nextflow workflow (e.g., TransPi) typically requires user specification of both a working directory as well as a final output (or ‘publish’) directory. The working directory contains all the intermediate files, including programmatic outputs that will become inputs to subsequent processes.

TransPi organizes the output files that have been designated in the workflow definition as ‘final products’ in well-defined subdirectories indicative of their contents (for example draft and final assemblies). The complete output from the TransPi workflow is quite extensive, and as such is beyond the scope of a training module to explore completely. We instead chose to give users the skills to download the complete directory to their local machine for more complete investigation offline on their own schedule.

When running the workflow using compute resources within Vertex AI (Submodule 02 and 03), all storage is local to the instance. When run using Google Batch (Submodule 04), the published output is written to a designated GCP storage bucket. We provide instructions in both situations for the user to download the complete final output. For files and directories that are stored within a GCP bucket, we provide descriptions of how to use GCP’s command-line interface, specifically *gsutil*, to copy the files and directories to any other machine, whether that be an external machine or a Vertex AI instance.

## Discussion and conclusions

This module is designed to provide researchers with a basic understanding of transcriptome assembly and the novel research questions that can be asked using TransPi not only as a primary analysis tool but also as a first step leading to further exploration such as differential expression that is afforded by an annotated transcriptome.

In addition, this module provides the foundational knowledge and skills to understand computing workflow and container systems, two technologies critical to modern computational biology and Cloud computing. We specifically introduce module users to the Nextflow workflow/pipeline system and Docker containers. Working within these systems facilitates not only reduces the barriers of entry into a new type of data analysis but it also enforces documented, reproducible data analysis.

Critically, while this module is designed to be run with modest computing resources and cost, the skills imparted should subsequently allow interested researchers to apply the principles to larger problems of interest.

Development of this module was a joint effort that required the biological and bioinformatics knowledge of the MDIBL research team, paired with the technical Cloud computing knowledge of the combined Deloitte and Google teams. Absent that collaboration, mapping the computational needs of assembly onto the Google compute platform would have certainly required significantly longer time and effort on the part of the MDIBL team.

Pairing the benefits of Nextflow with GCP and Google Batch to start and stop Cloud computing resources for specific processes provides the research community, especially those in the NIH IDeA program unprecedented access to complex analysis protocols in a cost-effective manner. By using Nextflow in the context of Google Batch, researchers only pay for what resources as long as they are needed, saving both money and time, with minimal barriers to entry. Ultimately, Cloud computing is changing the ways researchers are handling their data, and new innovations such as Nextflow and open-source communities such as nf-core [21] afford users a streamlined process to get them that much closer to the answers to their questions.

Note that Google Cloud is an evolving technology that has regular updates. This learning module is actively being maintained on GitHub and as these updates happen, the module will be revised with technical changes to work with the current state of Google Cloud along with new material.

**Key Points**

- The NIH STRIDES program has facilitated the development of a series of training modules in using Cloud computing resources.
- Cloud computing represents a new paradigm that allows researchers to carry out large-scale analysis.
- Use of Cloud resources requires updated knowledge and skills.
- We present a module that uses the biological problem of transcriptome assembly to teach concepts of:
  - Cloud computing,
  - workflow/pipeline management, and
  - computing container systems.
- Our module provides an introduction to use of the Google Batch Application Programming Interface (API), which
  - automatically controls the use of all necessary resources
  - enables cost- and resource-efficient use of Cloud computing.

## Data Availability

All training materials from this module can be obtained at the GitHub repository: https://github.com/NIGMS/Transcriptome-Assembly-Refinement-and-Applications. The underlying raw data can be obtained through the NIH SRA and TSA resources.

## References

[1] Slatko BE , Gardner AF, Ausubel FM. Overview of next generation sequencing technologies. Curr Protoc Mol Biol 2018;122:e59. 10.1002/cpmb.59. 29851291 PMC6020069

[2] Conesa A , Madrigal P, Tarazona S. et al. A survey of best practices for RNA-seq data analysis. Genome Biol 2016;17:13. 10.1186/s13059-016-0881-8. 26813401 PMC4728800

[3] Raghavan V , Kraft L, Mesny F. et al. A simple guide to de novo transcriptome assembly and annotation. Brief Bioinform 2022;23:bbab563. 10.1093/bib/bbab563. 35076693 PMC8921630

[4] Grabherr MG , Haas BJ, Yassour M. et al. Full-length transcriptome assembly from RNA-Seq data without a reference genome. Nat Biotechnol 2011;29:644–52. 10.1038/nbt.1883. 21572440 PMC3571712

[5] Schulz MH , Zerbino DR, Vingron M. et al. Oases: robust de novo RNA-seq assembly across the dynamic range of expression levels. Bioinformatics 2012;28:1086–92. 10.1093/bioinformatics/bts094. 22368243 PMC3324515

[6] Xie Y , Wu G, Tang J. et al. SOAPdenovo-trans: de novo transcriptome assembly with short RNA-Seq reads. Bioinformatics 2014;30:1660–6. 10.1093/bioinformatics/btu077. 24532719

[7] Bushmanova E , Antipov D, Lapidus A. et al. rnaSPAdes: a de novo transcriptome assembler and its application to RNA-Seq data. Gigascience 2019;8:giz100. 10.1093/gigascience/giz100. 31494669 PMC6736328

[8] Robertson G , Schein J, Chiu R. et al. De novo assembly and analysis of RNA-seq data. Nat Methods 2010;7:909–12. 10.1038/nmeth.1517. 20935650

[9] Hölzer M , Marz M. De novo transcriptome assembly: a comprehensive cross-species comparison of short-read RNA-Seq assemblers. Gigascience 2019;8:giz039. 10.1093/gigascience/giz039. 31077315 PMC6511074

[10] Rivera-Vicéns RE , Garcia-Escudero CA, Conci N. et al. TransPi—a comprehensive TRanscriptome ANalysiS PIpeline for de novo transcriptome assembly. Mol Ecol Resour 2022;22:2070–86. 10.1111/1755-0998.13593. 35119207

[11] Voshall A , Behera S, Li X. et al. A consensus-based ensemble approach to improve transcriptome assembly. BMC Bioinformatics 2021;22:513. 10.1186/s12859-021-04434-8. 34674629 PMC8532302

[12] DI Tommaso P , Chatzou M, Floden EW. et al. Nextflow enables reproducible computational workflows. Nat Biotechnol 2017;35:316–9. 10.1038/nbt.3820. 28398311

[13] Waterhouse RM , Seppey M, Simao FA. et al. BUSCO applications from quality assessments to gene prediction and Phylogenomics. Mol Biol Evol 2018;35:543–8. 10.1093/molbev/msx319. 29220515 PMC5850278

[14] Simão FA , Waterhouse RM, Ioannidis P. et al. BUSCO: assessing genome assembly and annotation completeness with single-copy orthologs. Bioinformatics 2015;31:3210–2. 10.1093/bioinformatics/btv351. 26059717

[15] Manni M , Berkeley MR, Seppey M. et al. BUSCO: assessing genomic data quality and beyond. Curr Protoc 2021;1:e323. 10.1002/cpz1.323. 34936221

[16] Hartig EI , Zhu S, King BL. et al. Cortisol-treated zebrafish embryos develop into pro-inflammatory adults with aberrant immune gene regulation. Biol Open 2016;5:1134–41. 10.1242/bio.020065. 27444789 PMC5004618

[17] Torres-Sánchez M , Creevey CJ, Kornobis E. et al. Multi-tissue transcriptomes of caecilian amphibians highlight incomplete knowledge of vertebrate gene families. DNA Res 2019;26:13–20. 10.1093/dnares/dsy034. 30351380 PMC6379020

[18] Wang J , Fu L, Koganti PP. et al. Identification and functional prediction of large intergenic noncoding RNAs (lincRNAs) in rainbow trout (*Oncorhynchus mykiss*). Mar Biotechnol (NY) 2016;18:271–82. 10.1007/s10126-016-9689-5. 26864089

[19] Al-Tobasei R , Paneru B, Salem M. Genome-wide discovery of long non-coding RNAs in rainbow trout. PloS One 2016;11:e0148940. 10.1371/journal.pone.0148940. 26895175 PMC4764514

[20] Salem M , Paneru B, Al-Tobasei R. et al. Transcriptome assembly, gene annotation and tissue gene expression atlas of the rainbow trout. PloS One 2015;10:e0121778. 10.1371/journal.pone.0121778. 25793877 PMC4368115

[21] Ewels PA , Peltzer A, Fillinger S. et al. The nf-core framework for community-curated bioinformatics pipelines. Nat Biotechnol 2020;38:276–8. 10.1038/s41587-020-0439-x. 32055031

